# Potential Amoebicidal Activity of Hydrazone Derivatives: Synthesis, Characterization, Electrochemical Behavior, Theoretical Study and Evaluation of the Biological Activity

**DOI:** 10.3390/molecules20069929

**Published:** 2015-05-29

**Authors:** Yanis Toledano-Magaña, Juan Carlos García-Ramos, Marisol Navarro-Olivarria, Marcos Flores-Alamo, Mayra Manzanera-Estrada, Luis Ortiz-Frade, Rodrigo Galindo-Murillo, Lena Ruiz-Azuara, Ruth Ma. Meléndrez-Luevano, Blanca M. Cabrera-Vivas

**Affiliations:** 1 Departamento de Inmunología, Instituto de Investigaciones Biomédicas, Universidad Nacional Autónoma de México, Avenida Universidad 3000, 04510 Mexico City, Mexico; E-Mail: yanistoledano@gmail.com; 2 Laboratorio de Química Inorgánica Medicinal, Departamento de Química Inorgánica y Nuclear, Facultad de Química, Universidad Nacional Autónoma de México, Avenida Universidad 3000, 04510 Mexico City, Mexico; E-Mails: johnycarbonilo@gmail.com (J.C.G.-R.); lenar701@gmail.com (L.R.-A.); 3 Departamento de Química Orgánica, Facultad de Ciencias Químicas, Benemérita Universidad Autónoma de Puebla, 72570 Puebla, Mexico; E-Mails: marisol-navarro@hotmail.com (M.N.-O.); ruthmelendrez@gmail.com (R.M.M.-L.); 4 Facultad de Química, Universidad Nacional Autónoma de México, Avenida Universidad 3000, 04510 Mexico City, Mexico; E-Mail: mfa@unam.mx; 5 Departamento de Electroquímica, Centro de Investigación y Desarrollo Tecnológico en Electroquímica, S.C., Parque Tecnológico Querétaro, Sanfandila, Pedro de Escobedo, C.P. 76703 Querétaro, México; E-Mails: aryamm@hotmail.com (M.M.-E.); lortiz@cideteq.mx (L.O.-F.); 6 Department of Medicinal Chemistry, College of Pharmacy, University of Utah, 2000 East 30 South Skaggs 201, Salt Lake City, UT 84112, USA; E-Mail: rodrigogalindo@gmail.com

**Keywords:** hydrazone, amoebicidal activity, ROS production, electrochemistry, DFT, hydrogen bonds, *Entamoeba histolytica*

## Abstract

Four new hydrazones were synthesized by the condensation of the selected hydrazine and the appropriate nitrobenzaldehyde. A complete characterization was done employing ^1^H- and ^13^C-NMR, electrochemical techniques and theoretical studies. After the characterization and electrochemical analysis of each compound, amoebicidal activity was tested *in vitro* against the HM1:IMSS strain of *Entamoeba histolytica*. The results showed the influence of the nitrobenzene group and the hydrazone linkage on the amoebicidal activity. *meta-*Nitro substituted compound **2** presents a promising amoebicidal activity with an IC_50_ = 0.84 μM, which represents a 7-fold increase in cell growth inhibition potency with respect to metronidazole (IC_50_ = 6.3 μM). Compounds **1**, **3**, and **4** show decreased amoebicidal activity, with IC_50_ values of 7, 75 and 23 µM, respectively, as a function of the nitro group position on the aromatic ring. The observed differences in the biological activity could be explained not only by the redox potential of the molecules, but also by their capacity to participate in the formation of intra- and intermolecular hydrogen bonds. Redox potentials as well as the amoebicidal activity can be described with parameters obtained from the DFT analysis.

## 1. Introduction

Amoebiasis is caused by the protozoan parasite *Entamoeba hystolitica* and is globally considered a leading parasitic disease causative of human mortality [1]. About 10% of the world’s population is affected by amoebiasis and approximately 40,000–100,000 deaths per year are related to this protozoan, mainly in developing countries [2]. Acute amoebiasis presents symptoms such as diarrhea or dysentery with frequent and often bloody stools, whereas chronic amoebiasis can present gastrointestinal symptoms plus fatigue, weight loss and occasional fever. Extraintestinal amoebiasis can occur if the parasite spreads to other organs, most commonly the liver, where it causes amoebic liver abscesses with fever and right upper quadrant abdominal pain [3].

Metronidazole and other nitroimidazole compounds are effective for the treatment of invasive amoebiasis, but are less effective at eliminating parasites located in the intestinal lumen. Several side effects are reported, ranging from vomiting and diarrhea, hallucinations [4], encephalopathy [5,6], and cancer [7], to strains resistant to this drug [8]. Therefore, new and better alternative therapies to control amoebiasis are urgently required. To achieve this goal, several synthesized and naturally occurring compounds have been tested, yet there is no drug considered to be ideal for the treatment of amoebiasis, particularly for the treatment of severe infections [9]. In recent years, several hydrazone derivatives have shown notable results in this context, with IC_50_ values in the µM range [10,11,12] and in some cases with lower values than the drug of first choice for human amoebiasis treatment, metronidazole.

Besides their amoebicidal activity, several studies have demonstrated that hydrazones present antidepressant [13,14], anticonceptive [15], and anticancer activity [16]. Some derivatives have been assessed as antimicrobial [17] and bactericidal agents [18], showing promising results against *Mycobacterium tuberculosis* [19] as well as in other parasitic diseases such as leishmaniasis [20] and Chagas disease [21].

In this work we reported the synthesis, characterization, electrochemical behavior, theoretical study and the amoebicidal activity of four new hydrazones that possess a nitrobenzyl group: (*E*)-2-nitrobenzaldehyde phenylhydrazone (**1**), (*E*)-3-nitrobenzaldehyde phenylhydrazone (**2**), (*E*)-4-nitrobenzaldehyde phenylhydrazone (**3**) and (*E*)-2,4-dinitrobenzaldehyde phenylhydrazone (**4**). The aim of this study was to evaluate the effect of the nitro group position on the electronic and physicochemical properties as well as its consequences on the amoebicidal activity of these compounds.

## 2. Results and Discussion

**Scheme 1:**
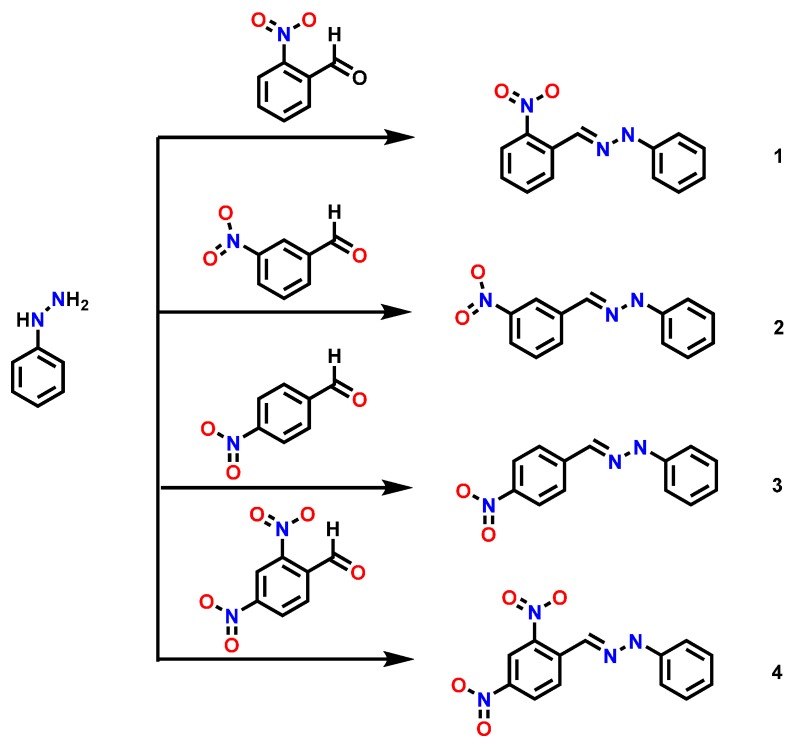
General synthesis of studied hydrazones.

The synthesis of hydrazones derivatives consisted in the condensation reaction of phenylhydrazine with 2-nitrobenzaldehyde (for compound **1**), 3-nitrobenzaldehyde (for **2**), 4-nitrobenzaldehyde (for **3**) and 2,4-dinitrobenzaldehyde (for **4**) (Scheme 1). Detailed reaction conditions can be found in our previous works [12,22]. The obtained products present intense red colors derived from the presence of a nitro group in the molecule, which produces absorption maxima in the UV and visible region of the electronic spectra that are easily followed.

The chemical shifts of the azomethine group of the studied hydrazones show the effect of the nitro group position on the electronic environment of the whole molecule. Substitution in the *ortho-*position produces the highest deshielding effect on the hydrogen of the azomethine group (hydrogen **e** of Scheme 1) as can be observed in the chemical shifts of compounds **1** (8.23 ppm) and **4** (8.63 ppm), followed by substitution in the *para-* (**3**, 7.94 ppm) and *meta-*position (**2**, 7.85 ppm), respectively (Table 1). The electronic communication between both aromatic rings has been established analyzing the chemical shift of the hydrazone moiety hydrogens (**d**) and hydrogen atoms **a**–**c**, where the resonance effect is patent due to the nitro group substitution in the *ortho-* and *para-* positions of one of the rings. The nitro group position effect on the electronic density distribution in the molecule is also observed in their electrochemical behavior, discussed below.

**Table 1:** Chemical shift of hydrogen atoms in the studied hydrazones.

Chemical Shift	1	2	3	4
a	6.87	6.85	6.86	6.82
b	7.27	7.27	7.26	7.26
c	7.12	7.14	7.21	7.13
d	9.15	8.98	10.0	11.42
e	8.23	7.85	7.94	8.63
f	--	8.98	7.88	--
g	8.19	--	8.22	8.09
h	7.44	8.04	--	--
i	7.64	7.58	8.22	8.41
j	7.91	8.05	7.88	8.35

### 2.1. Electrochemistry

**Figure 1:**
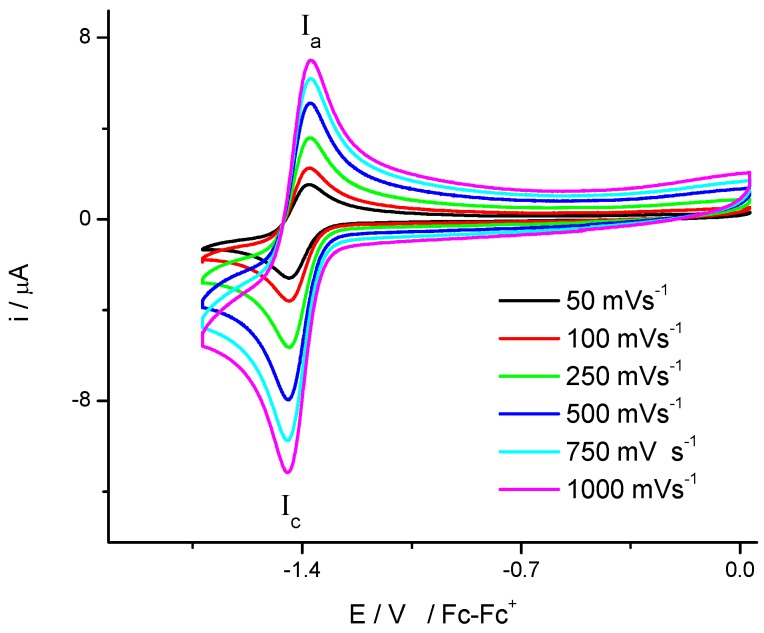
Compound **3** cyclic voltammogram in the presence of 0.1 M, tetrabutyl ammonium hexafluorophosphate (TBAPF_6_) in DMSO. Scan rate from 50 to 1000 mV·s^−1^. The working electrode used was platinum.

Figure 1 shows a series of voltammograms of compound **3**, at a scan rate from 50 mV·s^−1^ to 1000 mV·s^−1^, where one reduction reaction **I_c_** and its corresponding oxidation reaction **I_a_** can be observed. For both processes a linear relationship between the peak current I_pc_ and *v*^1/2^ was observed. The difference between the potential peak values Δ*E*p was close to 60 mV and independent of the scan rate, characteristic for a reversible one electron transfer [23]. The **I** process is attributed to the redox process R-NO_2_ + 1e → R-NO_2_^•−^ with a half wave potential value E_1/2_of −1.411 V/Fc-Fc^+^.

The electrochemical behaviour of compounds **1** and **2** are very similar to that observed for compound **3**. Changes in the values of half wave potential values (E_1/2_) are attributed to the position of the nitro group.

**Figure 2:**
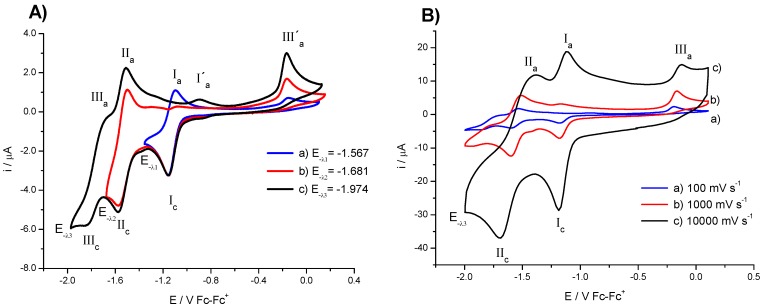
Cyclic voltammogram of compound **4** in the presence of 0.1 M TBAPF_6_ in DMSO, with scan rate of 100 mV·s^−1^. (**A**) The scan was initiated form E_i*=*0_ inverted at (a) E_λ1_ and at (b) E_λ2_ and (c) E_λ3_; (**B**) Behaviour of cyclic voltammograms at scan rate (a) 100 mV·s^−1^; (b) 1000 mV·s^−1^ and (c) 10,000 mV·s^−1^. The working electrode used was platinum.

In contrast to the discussed behavior, compound **4** presented a different electrochemical response, which can be understood with variable switching potential E_-λ_ experiments. When the potential scan was starting from open circuit potential and inverted at E_-λ1_, two oxidation signals (**III′_a_** and **I_a_**) and one reduction signal **I_c_** were detected (Figure 2A). The signals **I_a_** and **I_c_** correspond to the electron transfer R-NO_2_ + 1e → R-NO_2_^•−^, while signal **III′_a_** is related to a self-protonation processes of the electrogenerated radical anion [24,25]. On the other hand, when the potential scan was inverted at more negative values (E_-λ2_), two additional reduction and oxidation waves **II_c_** and **II_a_** were observed (see Figure 2A), which are associated to the a second electron transfer that produces the species ^−•^NO_2_-R-NO_2_^•−^. It can be also noticed that the signal **I_a_** is absent and the peak current for the signal **III′_a_** presents an increase in its value, due to the coupled self-protonation reactions mentioned before. In the case, where the switching potential was inverted at E_-λ3_, (Figure 2A) two new signals **III_c_** and **III_a_** were recorded, which are attributed to electroactive species that involves the formation of hydroxilammonium derivatives.

To confirm this idea an experiment at higher scan rate (10,000 mV·s^−1^) was carried out, see Figure 2B. On this time scale, the signals **I_a_** and **I_c_**, presented their corresponding oxidation signals **II_a_** and **II**c, and the current value of signals **III′_a_**, **III_c_** and **III_a_** shown a decrease. Thus, we conclude that the process **I** and **II** correspond to two consecutive processes of one electron transfer each one associated to the nitro moieties, see Equations (1) and (2), with half wave potential values (E_1/2_) that can be measured at high scan rate, where the chemical reaction is decoupled:

NO_2_-R-NO_2_ + 1e → NO_2_-R-NO_2_^•−^    **I** (1)

NO_2_-R-NO_2_^•−^ + 1e → ^−•^NO_2_-R-NO_2_^•−^    **II** (2)

The diffusion coefficients for all the compounds were calculated using the Randles-Sevcik equation in the forward scan considering the process **I_a_**. A summary of the main electrochemical parameters of all the compounds is presented in Table 2.

**Table 2:** Electronic parameters and half inhibition concentration (IC_50_) values expressed in µM for the nitrobenzene hydrazone compounds **1**–**4**.

Compound	E_1/2_ (I) (V) ^a^	E_1/2_ (II) (V) ^a^	Do (cm·s^−1^)	Total Energy ^d^	HOMO ^d^	LUMO ^d^	Energy Gap (H-L) ^d^	IC_50_ (µM)
**1**	−1.436 (−0.796) ^b^	n.o. ^c^	0.94 × 10^−5^	−816.045	−0.21008	−0.08477	−0.12531	75
**2**	−1.415 (−0.775) ^b^	n.o. ^c^	1.08 × 10^−5^	−816.050	−0.21551	−0.09123	−0.12428	0.84
**3**	−1.411 (−0.771) ^b^	n.o. ^c^	0.91 × 10^−5^	−816.051	−0.22004	−0.09413	−0.12591	7
**4**	−1.148 (−0.508) ^b^	−1.539 (−0.899)	0.81 × 10^−5^	−1020.45	−0.22572	−0.11294	−0.11278	23
**Metronidazole**	−0.486 ^b^							6.3

^a^ Half wave potential (E_1/2_) *vs.* Fc/Fc^+^ in the presence of 0.1 M TBAPF_6_ in DMSO; ^b^ Redox potential values were referenced to NHE employing E° = 0.640 V *vs.* NHE for Fc/Fc^+^ couple; ^c^ n.o. = not observed; ^d^ Electronic energy, orbital energy and energy gap expressed in Hartrees.

### 2.2. Computational Methods

To understand how the electronic density is distributed in the whole molecule and the variations caused by the different substituents, the molecular orbitals were calculated using DFT(m06-2x/6-344G(d,p)) and the electron density measurements were obtained under the QTAIM theory [26]. The theory of Frontier Molecular Orbitals is commonly used as an effective tool that aids in understanding bioactivity using electronic descriptors [27,28].

**Figure 3:**
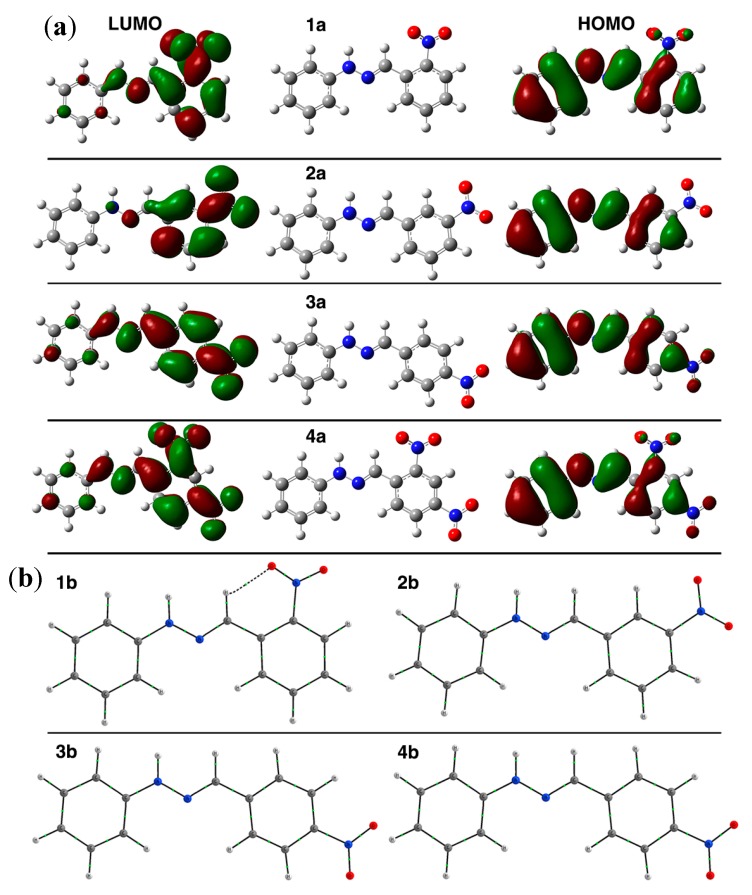
(**a**) Sketches for HOMO and LUMO orbitals of compounds **1**–**4**. The right side of the plot shows the LUMO orbital of the molecules; the left side shows the HOMO orbital; (**b**) QTAIM analysis showing the bond paths between the azomethine hydrogen atoms and the nitro groups.

**Figure 4:**
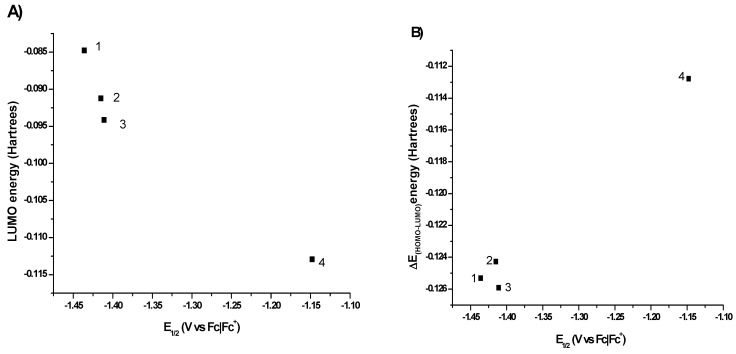

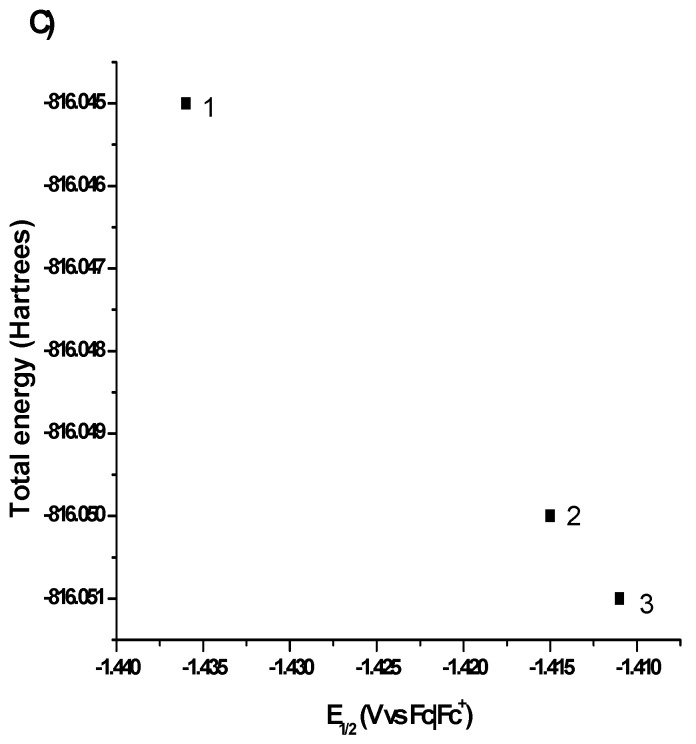
(**A**) Relationship found between the chemical shift of azomethine hydrogen (proton e) and the energy of the LUMO orbital; (**B**) Description of the redox potential value for the process R-NO_2_ + 1e → R-NO_2_^•−^ with ΔE_HOMO-LUMO_ values; (**C**) Change in the redox potential with the change in the total energy of the system

The results are shown in Table 2 and Figure 3. The energy of the orbital HOMO, LUMO and ΔE_HOMO-LUMO_ is expressed in Hartrees. The energy of each compound is presented in Table 2. We notice that the energy difference between the first three compounds is negligible (~0.005 Hartrees) which is expected since the only difference is the position of the NO_2_ group on the aromatic ring. The presence of a second NO_2_ group (*ortho* and *para*) in the same ring adds an extra 204.3 Hartrees to the total energy, as shown for compound **4**. In all cases the LUMO orbital is mainly located in the aromatic ring which possesses the nitro group, while the HOMO orbital is located in the whole molecule. In the case of the LUMO orbital for compound **1**, where the nitro group occupies the *ortho-*position of the aromatic ring, this orbital is located in the substituted aromatic ring, in the nitro group and in the hydrazone link with just a small participation of the non-substituted ring. For compound **2**, the location of LUMO orbital is substantially different and the orbital remains in the substituted ring and the hydrazone link has a limited contribution. Finally, for compounds **3** and **4** the LUMO orbital shows a similar distribution than the observed for compound **1**, as can be seen in Figure 4.

The energy of the LUMO orbital allows to explain the chemical shift of hydrogen **e** as can be seen in Figure 4A. The chemical shift of hydrogen **e** in compound **1** (8.23 ppm) could be due to the formation of an intramolecular hydrogen bond between the nitro group and the azomethine proton (Figure 3b), which produces a shift ca. 0.3 ppm higher than the same proton in the non-substituted structure, benzaldehyde phenylhydrazone (7.86 ppm) [29]. An enhanced effect is observed in hydrogen **e** of compound **4**, where the intramolecular hydrogen bond and a nitro group in a *para-*position with respect to the azomethine carbon exist. As the chemical shift is intrinsically related to the local electronic environment, these perturbations indicate a redistribution of the electron density upon H-bond formation. For H-bonding to an electronegative acceptor atom such as oxygen or nitrogen, there is always a change in the isotropic chemical shift of the H nucleus to higher frequencies (downfield shift). This downfield shift is a result of the decrease in the electron density around the hydrogen nucleus and deshielding effects from the electronic currents of the acceptor atom [30], which also has to produce a lower energy in the corresponding LUMO orbital.

In the case of the redox potential values found for the process R-NO_2_ + 1e → R-NO_2_^•−^, these values can be explained, as expected, with the difference of ΔE_HOMO-LUMO_ values (Figure 4B). The redox potential value for compound **1**, which presented the highest value of all the studied compounds and cannot be completely explained by the resonance or inductive effect exerted by the nitro group, can be explained due to the intramolecular hydrogen bond that forces the electron density to stay in the neighborhood and produce the employment of an extra energy to get the reduction process. For the mono-substituted systems (**1**–**3**), the best description of the redox potential was found with the total energy of the system as can be seen in Figure 4C.

### 2.3. X-ray Diffraction Studies

**Figure 5:**
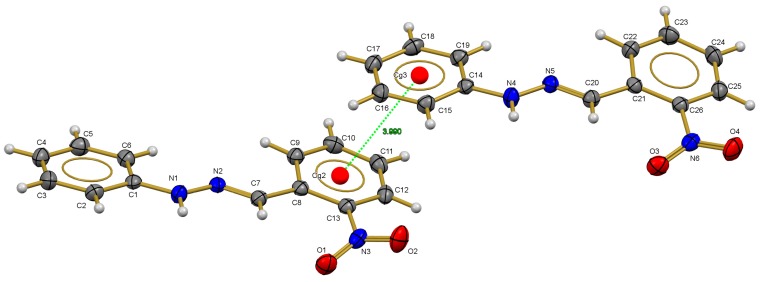
Crystal structure of compound **1** with the thermal ellipsoids drawn at 60% probability.

The asymmetric unit of compound **1** is formed by two molecules of (*E*)-1-(2-nitrobenzylidene)-2-phenylhydrazine (Figure 5), stabilized by π-π interaction with 3.990(9) Å of distance between the centroids cg2-cg3 (cg2 = C8-C9-C10-C11-C12-C13; cg3 = C14-C15 C16 C17 C18 C19); the planes formed by phenyl rings are planar with an angle of 12.50 (7) and 12.81 (7)° for the molecule 1 (C1 to C13) and molecule 2 (C14 to C26) in this compound. The C1-N1-N2-C7 (177.55 (12)°) and C8-C7-N2-N1 179.37 (12)°) torsion angles show evidence of the *E* configuration in the title compound.

**Figure 6:**
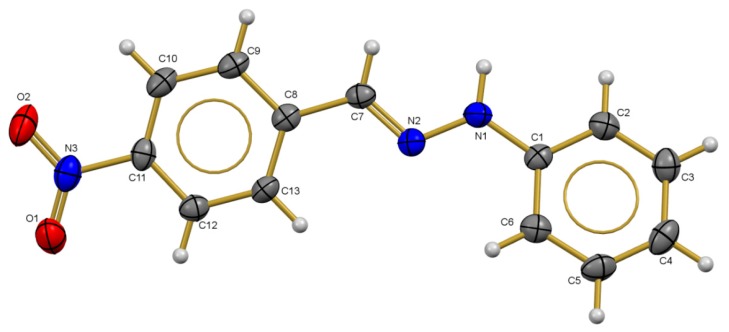
Crystal structure of compound **3** with the thermal ellipsoids drawn at 50% probability.

Similarly, the asymmetric unit of compound **3** consists of one molecule of (4-nitrobenzylidene)-2-phenylhydrazine (Figure 6) showing an *E* configuration on C=N group with phenylhydrazine group opposite to the nitrobenzylidene group. For the C1-N1-N2 (121.92 (17)°) angle in the Cambridge Structural Database (CSD), it was found that the angle is slightly larger than the mean (120.289°) value with σ = 1.19, while the C7-N2-N1 (115.18 (17)°) angle is very similar to the mean (116.14°) reported in CSD. The planes formed by phenyl rings are planar with an angle of 17.51 (9)°. The C1-N1-N2-C7 (172.63 (17) °) and C8-C7-N2-N1 (175.98 (16)°) torsion angles show evidence of the *E* configuration in the title compound.

In the crystalline structure there is one interaction of type N-H···O hydrogen bond between the N nitrogen donor atom and O acceptor atom. The hydrogen bond N1-H1D···O2 (2.08 (3)Å) with symmetry operation −x + 5/2, −y + 1, z + 1/2 and *C*_1_^1^(10) motif form an infinite one-dimensional chain with base vector [0,0,1], along the crystalline *c-axis*.

In summary, the electronic communication between the aromatic rings is manifested in the bond distances found in structures **1** and **4**. The main differences were found in the bond length of both aromatic rings with the substituent groups. On one side the *C_arom_-NO_2_ bond distance and on the other side, the N-^#^C_arom_ bond, where N-^#^C_arom_ distance (^#^C_arom_ = non-substituted ring) only three bonds have shown differences, that is, N-N, N-C_arom_ and C_arom_-NO_2_ bond lengths, listed in Table 3. The N-N bond lengths 1.347(2) and 1.344(2) Å for compounds **1** and **4** respectively, are shorter than the reported by Allen [28] (1.401 Å) for N-N bonds in planar structures, but similar to other hydrazone derivatives studied by our group (Table 3). Besides, the former bond length is shorter for N-^#^C_arom_ (^#^C_arom_ = non-substituted ring), the bond lengths are 1.385(2) Å (**1**) and 1.392(2) Å (**4**) are similar to those found in other hydrazone compounds (Table 3). However, it has to be noted that differences in these distances can be attributed to the position of the nitro group; the N=C double bond distances are slightly higher than the typical N=C bond distance (1.279(8) Å) reported by Allen [31]. The influence of the nitro group on the N-N distance is appreciated comparing the structure of *E*-benzaldehyde phenylhydrazone with the nitrobenzyl-derivatives, as in the former structure the N-N distance is shorter (1.330 Å) than that observed in the former compounds due to the electron withdrawing effect of the nitro group (Table 3). On the other hand, if the nitro group is present on the other aromatic ring ((*E*)-2-nitrobenzaldehyde-4-nitrophenylhydrazone) the N-N bond length gets larger, the same effect than that produced by the presence of two aromatic ring such as in *(E)-*1-(4-nitrobenzylidene)-2,2-diphenylhydrazine and *(E)-*1-(2,4-dinitrobenzylidene)-2,2-diphenylhydrazine (Table 3).

Finally, the bond distances of the calculated structures using DFT is presented in Table 3 which show a good agreement with the experimental structures. The RMSD value between the X-ray and the calculated structure is 0.19 Å and 0.20 Å for compounds **1** and **3**, respectively.

### 2.4. Amoebicidal Activity

In general, the amoebicidal activity of nitroimidazole derivatives is related to their capacity to generate reactive oxygen species (ROS) through the biotransformation of the nitro group that generates a nitroso free radical [32]. The referred biotransformation occurs through the electron transfer of ferredoxin or flavodoxin previously reduced by ferredoxin oxidoreductase to nitroimidazole derivatives [33]. Moreover, *Entamoeba histolytica* strains have developed resistance to treatment with nitroimidazole derivatives, associated in first instance to the down-regulation of pyruvate: ferredoxin oxidoreductase [34] and later related with the high levels of expression of iron containing superoxide dismutase (Fe-SOD) [35]. Thus, to generate compounds that can be able to produce an important redox imbalance in the parasite, it is fundamental to explore new molecules such as nitrohydrazone derivatives.

**Table 3:** Selected bond length (Å) and angles (°) for compounds **1**, **3** and similar compounds found in literature. Distances from the DFT calculations are shown in brackets.

Compound ^a^	^#^C_arom_-N	N-N	N=C	C-*C_arom_	*C_arom_-NO_2_	N-O	Ref.
**1**	1.407 (1.393)	1.341 (1.327)	1.286 (1.288)	1.450 (1.460)	1.489 (1.469)	1.219 (1.220), 1.216 (1.224)	This work
**3**	1.381 (1.394)	1.354 (1.327)	1.282 (1.286)	1.455 (1.451)	1.464 (1.463)	1.242 (1.221), 1.226 (1.222)	This work
*(E)-*1-(2-Nitrobenzylidene)-2,2-diphenylhydrazine	1.471	1.287	1.359	1.441	1.467	1.226, 1.227	[36]
*(E)-*1-(4-Nitrobenzylidene)-2,2-diphenylhydrazine	1.437	1.363	1.285	1.456	1.464	1.216, 1.218	[37]
*(E)-*1-(2,4-Dinitrobenzylidene)-2,2-diphenylhydrazine	1.434	1.353	1.289	1.459	1.468	1.219, 1.212	[38]
*(E)-*1-(Benzylidene)-2,2-diphenylhydrazine	1.441	1.369	1.279	1.469	------	------	[39]
*(E)-*Benzaldehyde phenylhydrazone	1.436	1.330	1.312	1.416	------	------	[40]
*(E)-*2-Nitrobenzaldehyde-4-nitrophenylhydrazone	1.369	1.355	1.283	1.467	1.471	1.227, 1.225	[41]
*(E)-*2-Nitrobenzaldehyde-4-nitrophenylhydrazone	1.371	1.351	1.275	1.464	1.471	1.215, 1.212	[42]
*(E)-*Benzaldehyde-2,4-dinitrophenylhydrazone	1.350	1.374	1.275	1.461	------	------	[43]

^a^ Average bond lengths in Å; ^#^C_arom_ = non-substituted ring; *C_arom_ = -NO_2_ substituted ring.

In a previous work [12] we established that the amoebicidal activity of hydrazone derivatives was related to two main factors: (i) the redox potential of groups susceptible to be reduced such as nitro or the double bond in the hydrazone and (ii) the availability of the hydrogen atom from the imine carbon to form hydrogen bond interactions or some other type of molecular recognition. In this work, it was found that the amoebicidal activity of hydrazones increases as the redox potential is closer to −0.480 V and is even better when the value is closer to zero, as in the case of 5-(4-nitrophenyl)-2-furaldehyde phenylhydrazone (−0.425 V, IC_50_ = 0.98 µM). However, the results of this work show that there is not a specific redox potential range around that observed for metronidazole to ensure a good amoebicidal activity.

The amoebicidal activity of compounds **1**–**4** expressed as IC_50_ in µM units are shown in Table 2. From the results obtained with these compounds it is evident the effect of the position of the nitro group over the observed amoebicidal activity. The better amoebicidal activity was found for compound **2** (*m*-nitro) with an IC_50_ value of 0.84 µM, followed by compound **3** (*p*-nitro) with an IC_50_ value of 7 µM and the nitro group in the *ortho*- position (**1**) presented the lowest amoebicidal activity with an IC_50_ = 75 µM. The dinitro derivative (compound **4**) presents an behavior intermediate between compounds **1** and **3**, with IC_50_ = 25 µM. The change of the nitro group from the *meta*-(**2**) to the *para*-(**3**) position on the aromatic ring produces an 8-fold decrease in the amoebicidal activity, while the presence of the nitro group in the *ortho*- position (**1**) generates a decrease in the amoebicidal activity of almost two orders of magnitude.

Interestingly, even though the great differences observed in the amoebicidal activity, only small differences were found in the redox potential values for the reduction of the nitro group. Besides that fact, the redox potential values found for these nitrohydrazone derivatives were ~0.3 V more negative than the value associated to metronidazole.

**Figure 7:**
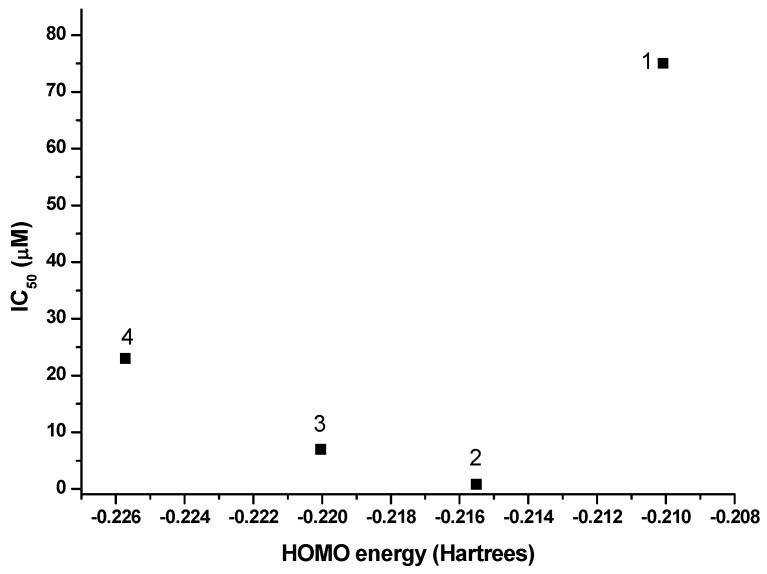
HOMO orbital energy effect over the amoebicidal activity (IC_50_) of nitrobenzene hydrazone derivatives.

This allow us to propose that there is not a redox potential range to ensure a good amoebicidal activity of the compounds, but the presence of nitro group enhances the amoebicidal activity with a strong dependence onits position as a substituent. The amoebicidal activity of these compounds can also be described by the change in the HOMO orbital energy—as the energy of this orbital decreases the amoebicidal activity is the lowest (Figure 7), the same tendency observed with the redox potential for the transformation of the nitro group. However, for the compound where the nitro group participates in the intra-molecular hydrogen bond (**1**) even though the energy of the system is the lowest of all the studied systems, the amoebicidal activity is the worst found. This allows us to suggest that the hydrogen atoms of the hydrazone must play an important role in the molecular recognition of these compounds by ferredoxin, flavodoxin or maybe DNA due to the great difference observed in the amoebicidal activity. Thus further studies have to be done in this direction.

## 3. Experimental Section

### 3.1. Synthesis and Characterization

Physical measurements were done employing the following equipments: UV-vis spectra were recorded on Varian Cary 50 Bio UV Visible spectrophotometer (Varian Instruments, Walnut Creek, CA, USA); IR (KBr) spectra were recorded on Nicolet FTIR Magna 750 (Nicolet Instrument Corporation, Canton, MA, USA); ^1^H-NMR (400 MHz) and ^13^C-NMR (100 MHz) spectra were obtained on Varian VX-400 spectrometer (Varian Inc., Palo Alto, CA, USA), chemical shifts were given on the delta scale as parts per million (ppm). High Resolution MS and mass spectra were recorded on JEOL JEM JMS-SX 102 a 70 eV, JEOL USA INC, Peabody, MA, USA; data is given in mass/charge units (*m*/*z*). Electrochemical determinations were done on a Biologic SP-50 potentiostat/galvanostat (BioLogic Science Instruments, Claix, France). X-ray diffraction data were collected with an Oxford Diffraction Gemini “A” diffractometer equipped with a CCD area detector (Agilent (2011) CrysAlis PRO., Agilent Technologies, Yarnton, UK. An Olympus BX51 fluorescence microscope (Olympus Optical Company Ltd., Tokyo, Japan) was employed in the viability measurements of *Entamoeba histolytica* trophozoites.

*(E)-1-(2-Nitrobenzylidene)-2-phenylhydrazine* (**1**). Red crystals; yield: 93% at 25 °C, mp.144–147 °C. FTIR (film): (cm^−1^): 3289 ν(N-H), 1527 ν(C=N), 1491 ν(NO_2_). ^1^H-NMR ((CD_3_)_2_CO: (δ/ppm, *J*/Hz): 9.15 (s, 1H, NH), 8.23 (s,1H, C=N), 8.19 (d, 1H, C3), 7.91 (d, 1H, C6), 7.64 (t, 1H, C5) 7.44 (t, 1H, C4), 7.27 (m, 2H, C3ʹ), 7.12 (d, 2H, C2ʹ), 6.87 (t, 1H, C4ʹ).^13^C-NMR ((CD_3_)_2_CO): (δ/ppm): 147.36 (C=N), 144.81 (C2), 132.92 (C5), 130.98 (C3), 130.41 (C1), 129.19 (C3*'*), 128.10 (C4), 127.15 (C1ʹ), 124.51 (C6), 120.16 (C4ʹ), 112.72 (C2ʹ). EI-HRMS: calculated for (C_13_H_11_N_3_O_2_), 241.0851; found, 241.0990. MS-EI: *m*/*z* = 241.25 M^+^.C_13_H_11_N_3_O_2_. UV-vis (acetone, λ (nm)): λ = 198 (18,060), 223 (9510), 243 (7590), 292 (4320), 339 (3870).

*(E)-1-(3-Nitrobenzylidene)-2-phenylhydrazine* (**2**). Red powder; yield: 93% at 25 °C, mp.116–117 °C. FTIR (film): (cm^−1^): 3288 ν(N-H), 1532 ν(C=N), 1468 ν( NO_2_). ^1^H-NMR ((CD_3_)_2_CO: (δ/ppm, *J*/Hz): 8.98 (s, 1H, NH), 8.44 (s, 1H, C2), 8.09, (dd, *J* = 2 Hz, 1H, C4), 8.05 (dd, *J* = 8 Hz, 1H,C6), 7.85 (s, 1H, C=N), 7.58 (t, *J* = 8 Hz, 1H, C5), 7.27 (t, *J* = 8 Hz, 2H, C3*'*), 7.14 (d, *J* = 8 Hz 2H, C2*'*), 6.85 (t, *J* = 1.08 Hz, 1H, C4*'*). ^13^C-NMR ((CD_3_)_2_CO): (δ/ppm):144.83 (C=N), 138.22 (C3), 133.89 (C1ʹ), 131.32 (C6), 129.84 (C1), 129.82 (C5), 129.14 (C3ʹ), 121.90 (C4), 119.83 (C2*'*), 119.72 (C2), 112.51 (C4*'*). EI-HRMS: calculated for (C_13_H_11_N_3_O_2_), 241.0851; found, 241.0793. MS-EI: *m*/*z* = 241.25 M^+^. C_13_H_11_N_3_O_2_. UV-Vis (acetonitrile, λ = nm, ε = M^−1^·cm^−1^): λ = 199(27,380), 224(19,280), 244(17,710), 289(15,720), 338(11,280), 410(9830).

*(E)-1-(4-Nitrobenzylidene)-2-phenylhydrazine* (**3**). Wine crystals; yield: 90% at 25 °C, mp.144–147 °C. FTIR (film): (cm^−1^): 3294 ν(N-H), 1597 ν(C=N), 1550 ν(NO_2_). ^1^H-NMR ((CD_3_)_2_CO: (δ/ppm, *J*/Hz): 10.00 (s, 1H, NH), 8.22 (d, *J* = 5.14, 2H, C3), 7.94 (s, 1H, C=N), 7.88 (d, *J* = 5.14 Hz 2H,C2), 7.26 (m, 2H, C3*'*), 7.21 (m, *J* = 1.24, 2H, C2*'*), 6.86 (t, *J* = 1.24, 1H, C4*'*). ^13^C-NMR ((CD_3_)_2_CO): (δ/ppm): 146.74 (C4), 144.70 (C1), 142.80 (C1*'*), 133.82 (C=N), 129.21 (C3*'*), 126.09 (C2), 123.90 (C3),120.28 (C4*'*), 112.78 (C2*'*). EI-HRMS: calculated for (C_13_H_11_N_3_O_2_), 241.0851; found, 241.0850. MS-EI: *m*/*z* = 241.25 M^+^. C_13_H_11_N_3_O_2_. UV-Vis (acetonitrile, λ = nm, ε = M^−1^·cm^−1^) = 199 (19,170), 241 (8290), 313(4300).

*(E)-1-(2,4-Dinitrobenzylidene)-2-phenylhydrazine* (**4**). Dark red crystals; yield: 92% at 25 °C, mp. 223–225 °C. FTIR (film): (cm^−1^): 3286 ν(N-H), 1591 ν(C=N), 1492 ν(NO_2_). ^1^H-NMR ((CD_3_)_2_CO: (δ/ppm, *J*/Hz): 11.43 (s, 1H, NH), 8.69 (s, 1H, C3), 8.38 (m, 2H, C5, C=N), 8.29 (d, 1H, C6, 7.28 (t, 2H, C3), 7.15 (d, 2H, C2-Ph), 6.89 (t, 1H, C4*'*). ^13^C-NMR ((CD_3_)_2_CO): (δ/ppm): 145.45 (C4), 145.06 (C2), 143.84 (C1*'*), 136.11 (C1), 129.53 (C3*'*), 128.97 (C6), 127.97 (C=N), 127.02 (C5), 121.31 (C4*'*), 120.90 (C3), 113.30 (C2*ʹ*). EI-HRMS: calculated for (C_13_H_10_N_4_O_4_), 286.0702; found, 286.0717. MS-EI: *m*/*z* = 286.24 M^+^. C_13_H_10_N_4_O_4_. UV-Vis (acetonitrile, λ = nm, ε = M^−1^·cm^−1^): λ = 199 (28,870), 224 (28,860), 244 (28,840), 289 (5270), 338 (4320).

All the spectra are included in the Supplementary Materials.

### 3.2. Electrochemistry

Electrochemical studies were performed using a 1 mM sample concentration of each compound in DMSO solution + 0.1 M tetra-*N*-butylammonium hexaflurophospate (TBAPF_6_). A typical three electrode array was employed; a platinum disk as working electrode, and a platinum wire as counter-electrode and, a silver wire as a pseudo reference electrode. All solutions were bubbled with nitrogen 5 min prior each measurement. Cyclic voltammetry experiments were initiated from open circuit potential (E_ocp_) to negative direction, using a range of scan rate from 0.1 to 1 V·s^−1^.Current interrupt method was used for *iR* compensation during all the experiments. All potentials were reported *vs.* the couple Fc/Fc^+^ according to IUPAC convention [44].

### 3.3. X-ray Crystallography. Experimental Part

Crystals of compounds **1** and **3** mounted on glass fiber were studied with Oxford Diffraction Gemini “A” diffractometer with a CCD area detector (λ_MoKα_ = 0.71073 Å, monochromator: graphite) source equipped with a sealed tube X-ray source. Unit cell constants were determined with a set of 15/3 narrow frame/runs (1° in ω) scans. The data sets consisted of 149 and 126 frames of intensity data collected for **1** and **3**, respectively, with a frame width of 1° in ω, a counting time of 16 s/frame, and a crystal-to-detector distance of 55.00 mm. The double pass method of scanning was used to exclude any noise. The collected frames were integrated by using an orientation matrix determined from the narrow frame scans.

CrysAlisPro and CrysAlis RED software packages [45] were used for data collection and data integration. Analysis of the integrated data did not reveal any decay. Final cell constants were determined by a global refinement of 4042 and 2233 reflections (θ < 29°) for **1** and **3**, respectively. Collected data were corrected for absorbance by using analytical numeric absorption correction [46] using a multifaceted crystal model based on expressions upon the Laue symmetry using equivalent reflections. Structure solution and refinement were carried out with the program(s): SHELXS97 [47]; SHELXL97; and the software used to prepare material for publication: WinGX v2013.3 [48].

Full-matrix least-squares refinement was carried out by minimizing (*Fo*^2^ *− Fc*^2^)^2^. All non-hydrogen atoms were refined anisotropically. The H atoms of the amine group (H-N) were located in a difference map and refined isotropically with *Uiso*(H) = 1.2*Ueq* (N). H atoms attached to C atoms were placed in geometrically idealized positions and refined as riding on their parent atoms, with C-H = 0.93–0.95 Å with *U_iso_*(H) = 1.2*U_eq_*(C) for aromatic and methyne groups. Crystal data and experimental details of the structure determination are listed in Table 4.

Crystallographic data have been deposited at the Cambridge Crystallographic Data Center as supplementary material number CCDC 1050239-1050240 for compounds **1** and **3**. Copies of the data can be obtained free of charge via http://www.ccdc.cam.ac.uk/conts/retrieving.html (or from the CCDC, 12 Union Road, Cambridge CB2 1EZ, UK; Fax: +44 1223 336033; E-mail: deposit@ccdc.cam.ac.uk)

**Table 4:** Crystal data and structure refinement for **1** and **3** compounds.

Identification Code	1	3
Empirical formula	C_13_ H_11_ N_3_ O_2_	C_13_ H_11_ N_3_ O_2_
Formula weight	241.25	241.25
Temperature	130(2) K	298(2) K
Wavelength	0.71073 Å	0.71073 Å
Crystal system	Orthorhombic	Orthorhombic
Space group	P b c a	P 21 21 21
Unit cell dimensions	a = 19.0466(8) Å	a = 6.0462(11) Å
	b = 12.0301(6) Å	b = 11.3924(17) Å
	c = 19.8489(7) Å	c = 17.004(3) Å
Volume	4548.0(3) Å3	1171.3(3) Å3
Z	16	4
Density (calculated)	1.409 Mg/m^3^	1.368 Mg/m^3^
Absorption coefficient	0.099 mm^−1^	0.096 mm^−1^
F(000)	2016	504
Crystal size	0.57 × 0.35 × 0.17 mm^3^	0.40 × 0.29 × 0.22 mm^3^
Theta range for data collection	3.539 to 29.501°	3.576 to 29.499°
Index ranges	−26 ≤ h ≤ 24, −9 ≤ k ≤ 15, −27 ≤ l ≤ 18	−8 ≤ h ≤ 7, −10 ≤ k ≤ 15, −15 ≤ l ≤ 22
Reflections collected	16711	3932
Independent reflections	5478 [R(int) = 0.0300]	2571 [R(int) = 0.0226]
Completeness to theta = 25.242°	99.8%	99.8%
Refinement method	Full-matrix least-squares on F2	Full-matrix least-squares on F2
Data/restraints/parameters	5478/0/331	2571/0/166
Goodness-of-fit on F2	1.017	1.094
Final R indices [I > 2 sigma(I)]	R1 = 0.0444, wR2 = 0.0925	R1 = 0.0389, wR2 = 0.0868
R indices (all data)	R1 = 0.0736, wR2 = 0.1055	R1 = 0.0448, wR2 = 0.0929
Largest diff. peak and hole	0.232 and −0.249 e.Å^−3^	0.149 and −0.231 e.Å^−3^

### 3.4. Amoebicidal Activity

#### 3.4.1. Parasite culture 

*E. histolytica* HM1-IMSS trophozoites were axenically grown in TYI-S33 medium [49]. Amoebas (1 × 10^5^ of live trophozoites) were placed in tubes with 3 mL TYI-S33 supplemented with each compound so that the final concentrations were as follows: 1000, 100, 10, 1, 0.1 and 0.01 μM.

#### 3.4.2. Parasite Viability

Amoebic trophozoite viability was assessed employing two different methods, (1) vital marker Trypan blue and (2) carboxyfluorescein diacetate (CFDA) and propidium iodide done at 24, 28 and 72 h under a microscope using a haemocytometer. In brief, samples of 100 μL containing around 1 × 10^4^ treated parasites were added with 100 μL Trypan blue 0.4% or 1 μL of 5 μM CFDA (Molecular Probes, Inc., Eugene, OR, USA) and 1 μL of 1.5 μM propidium iodide, mixed and incubated at room temperature for 15 min. Viable cells were counted using the Olympus BX51 fluorescence microscope.

## 4. Conclusions

The nitrophenyl hydrazone derivatives studied in this work have shown the importance of the redox potential of the nitro group and the availability of hydrogen bonds in their potential amoebicidal activity. There is no need to reach a redox potential near that observed for metronidazole to get good amoebicidal activity, but the presence of a nitro group in the *meta*- or *para*- position of the aromatic ring enhances this activity. Additionally, the availability of the hydrogen atoms seems to be an important factor to produce a better amoebicidal activity. The amoebicidal activity of these compounds can be described employing the HOMO energy values obtained from a DFT study, thus, this strategy should be explored with other compounds. Finally, it is important to know the mechanism of action of these kinds of compounds to provide a new lead family of molecules in the treatment of this parasitic disease and other diseases that could be susceptible to the same activity mechanism.

## Supplementary Materials

Supplementary materials can be accessed at: http://www.mdpi.com/1420-3049/20/06/9929/s1.
