# Supplementary material for: Potential Amoebicidal Activity of Hydrazone Derivatives: Synthesis, Characterization, Electrochemical Behavior, Theoretical Study and Evaluation of the Biological Activity
Source: Molecules. 2015 May 29;20(6):9929–48. doi: 10.3390/molecules20069929 (PMC6272681; doi:10.3390/molecules20069929)
Supplement: Supplementary file 1 [file molecules-20-09929-s001.pdf]

# Supplementary Materials

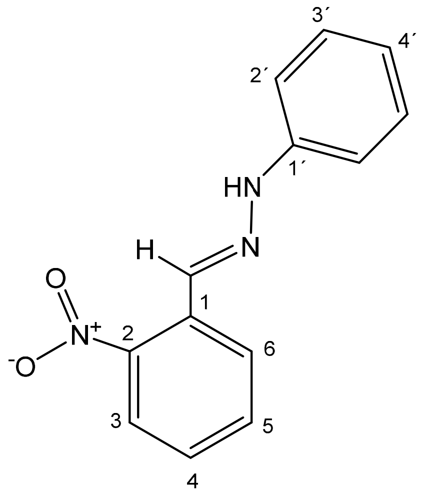


*(E)*-1-(2-nitrobenzylidene)-2-phenylhydrazine

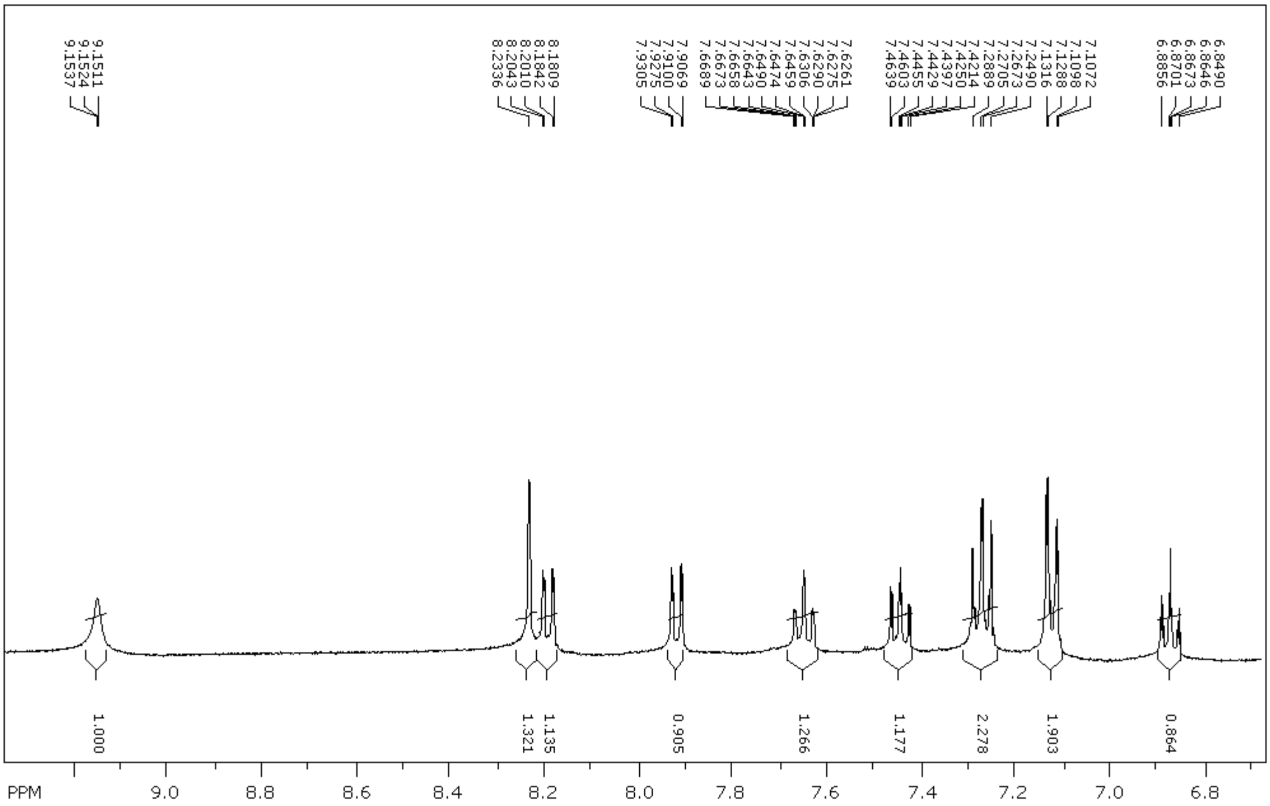


**Figure S1.** $^{1}$H-NMR for *(E)*-1-(2-nitrobenzylidene)-2-phenylhydrazine (compound **1**).

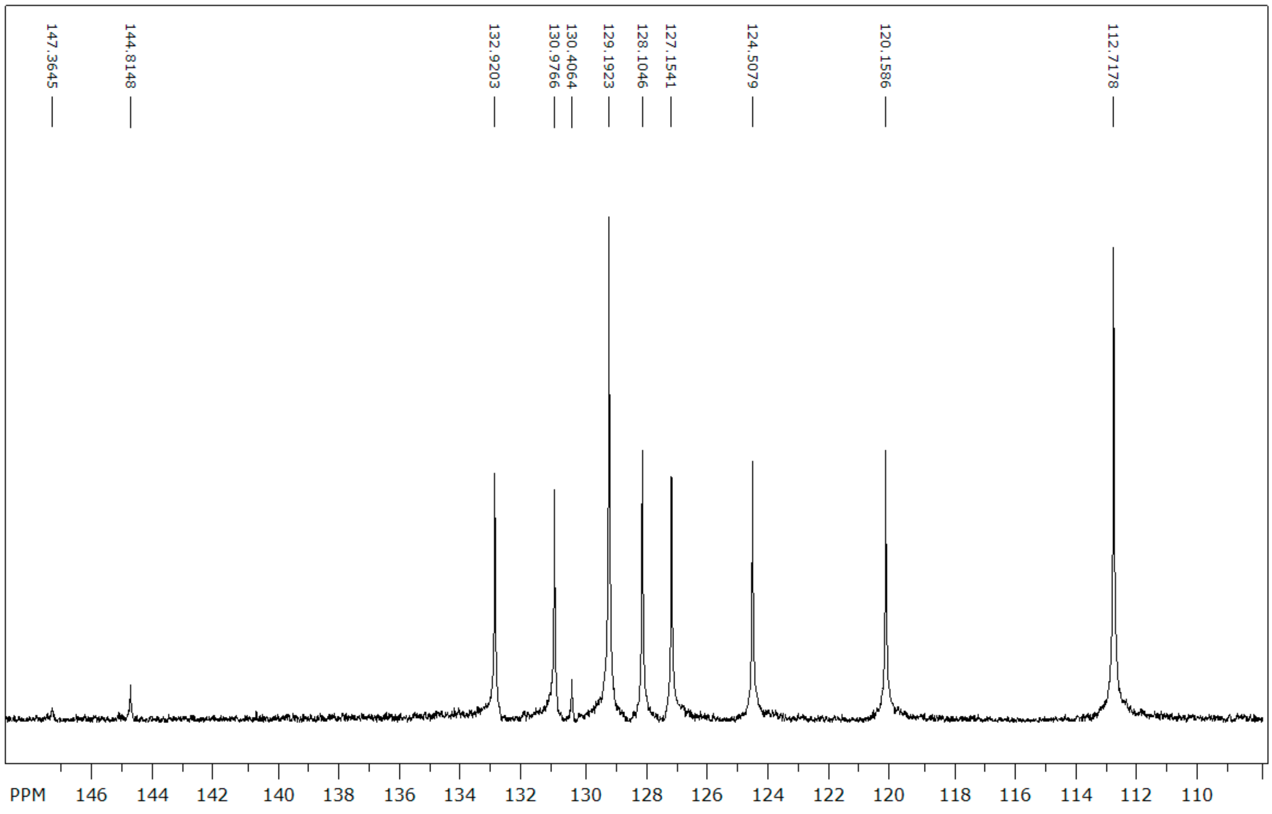


**Figure S2.** $^{13}$C-NMR for *(E)*-1-(2-nitrobenzylidene)-2-phenylhydrazine (compound **1**).

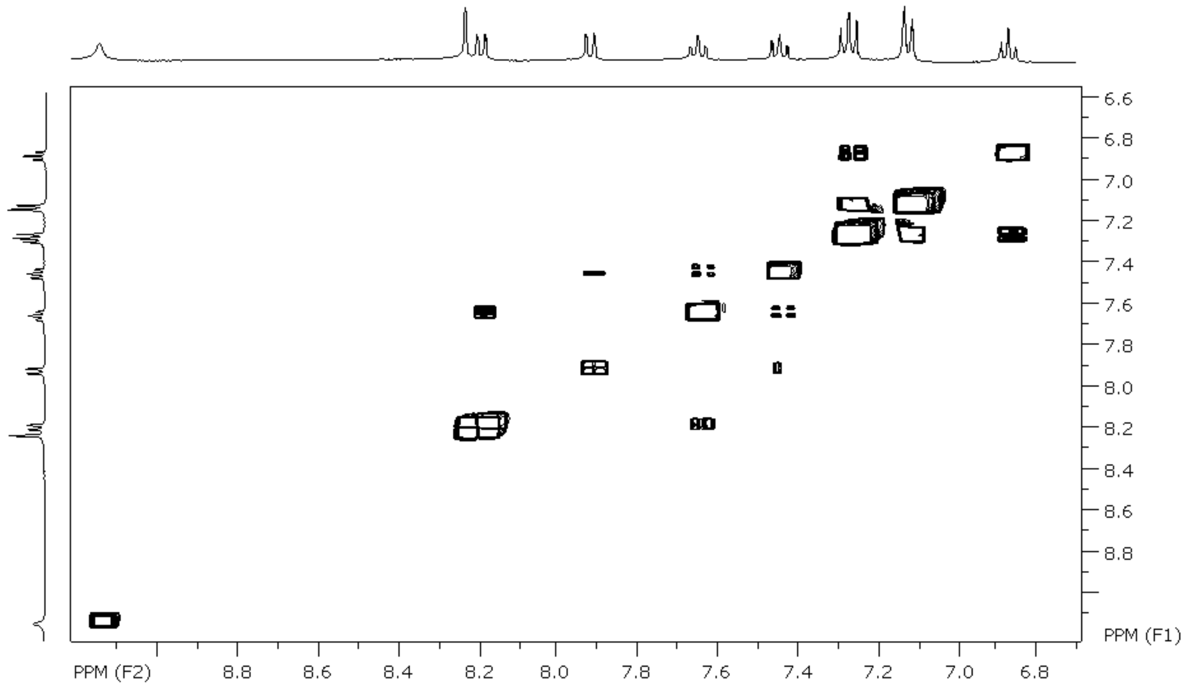


**Figure S3.** COSY for *(E)*-1-(2-nitrobenzylidene)-2-phenylhydrazine (compound **1**).

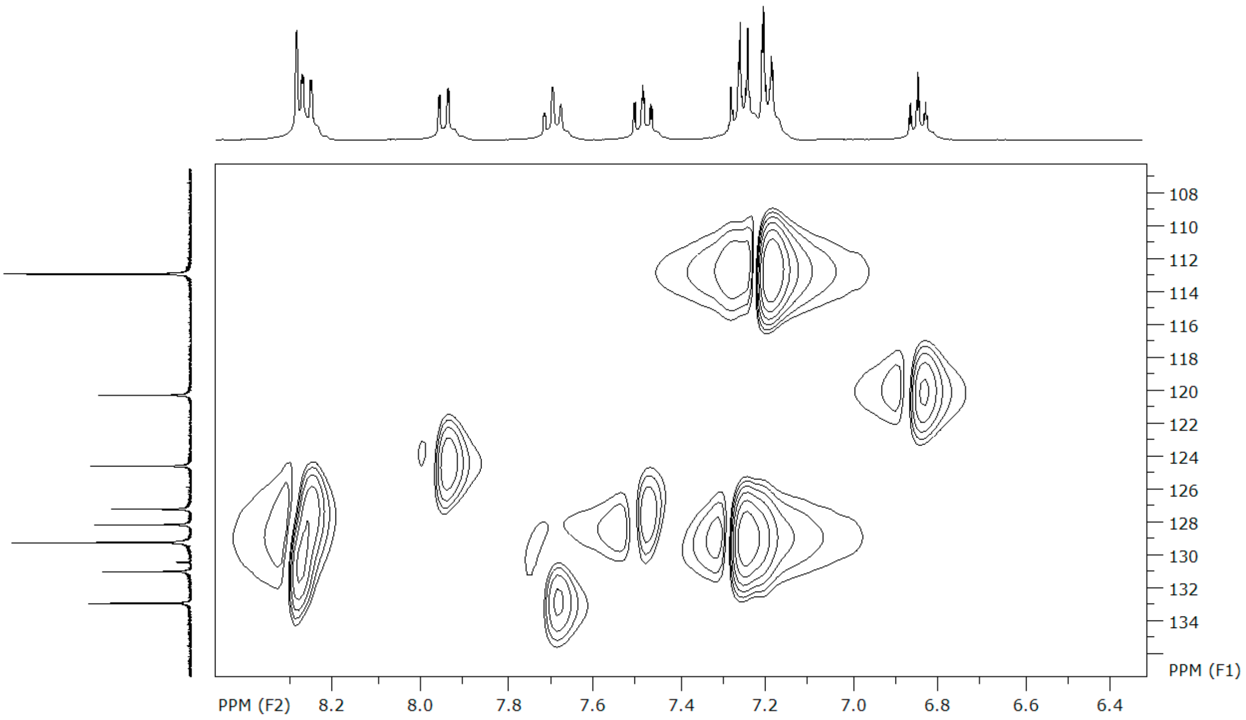


**Figure S4.** HSQC for *(E)*-1-(2-nitrobenzylidene)-2-phenylhydrazine (compound **1**).

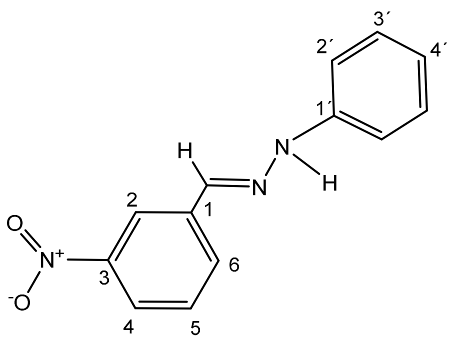


*(E)*-1-(3-nitrobenzylidene)-2-phenylhydrazine

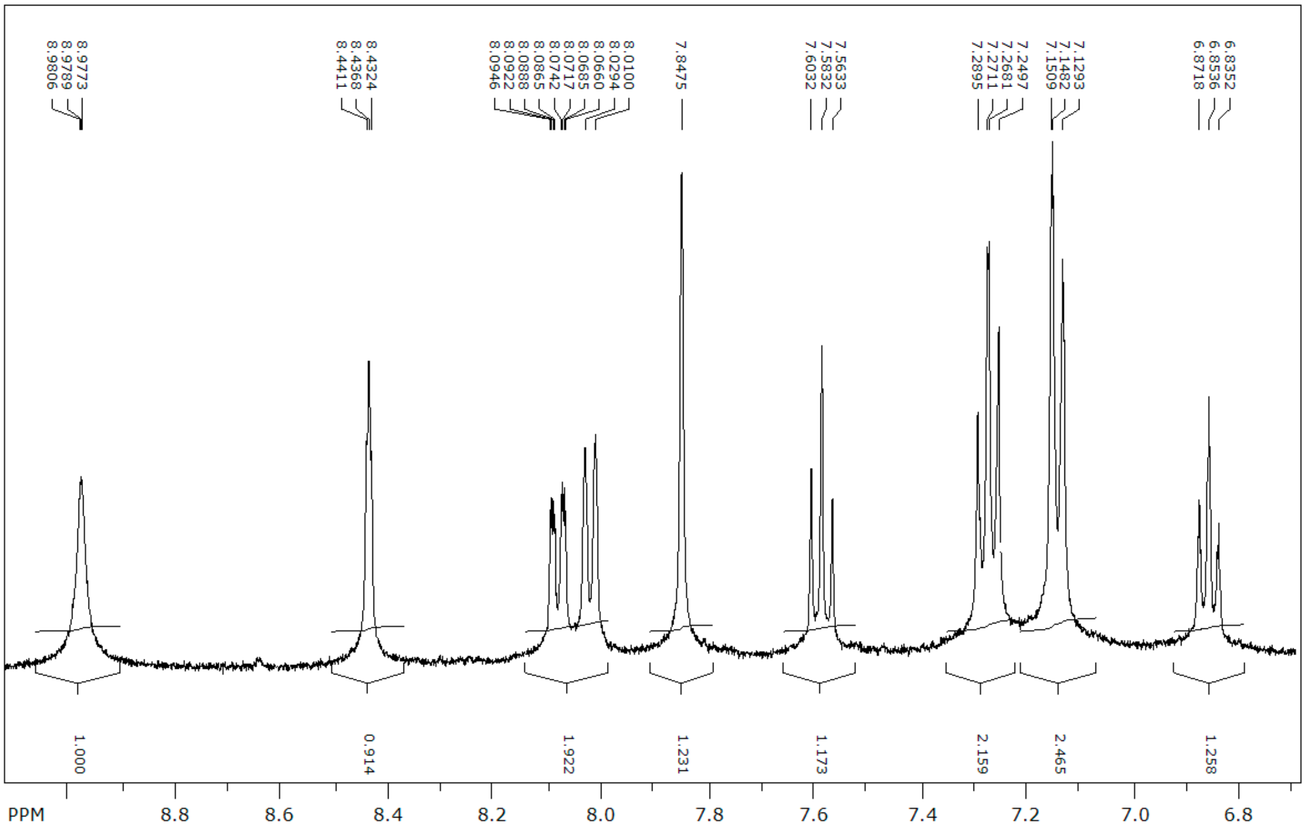


**Figure S5.** $^{1}$H-NMR for *(E)*-1-(3-nitrobenzylidene)-2-phenylhydrazine (compound **2**).

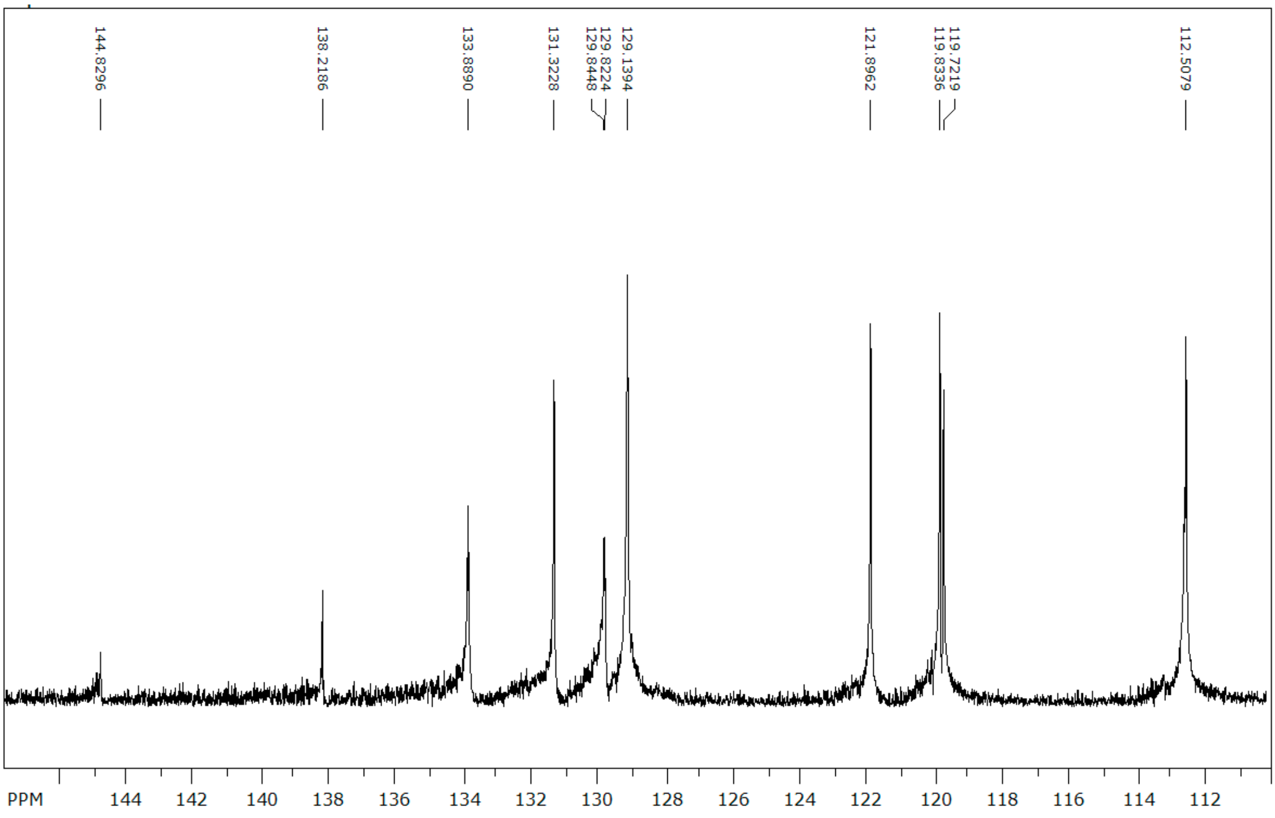


**Figure S6.** $^{13}$C-NMR for *(E)*-1-(3-nitrobenzylidene)-2-phenylhydrazine (compound **2**).

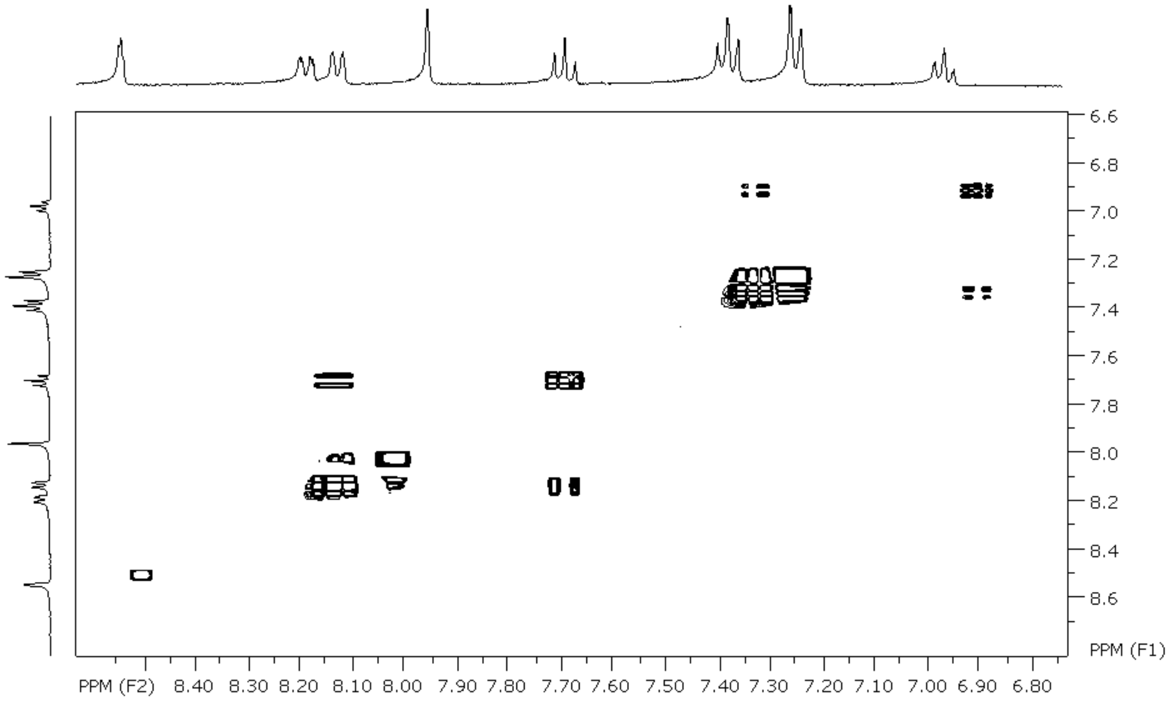


**Figure S7.** COSY for *(E)*-1-(3-nitrobenzylidene)-2-phenylhydrazine (compound **2**).

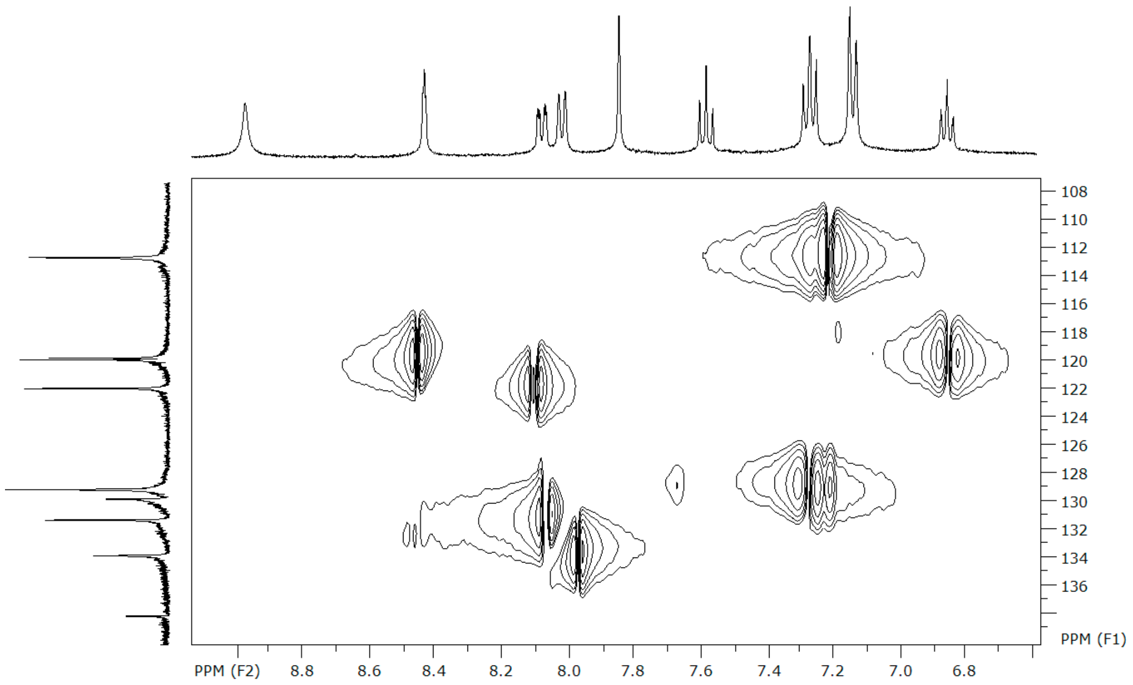


**Figure S8.** HSQC for *(E)*-1-(3-nitrobenzylidene)-2-phenylhydrazine (compound **2**).

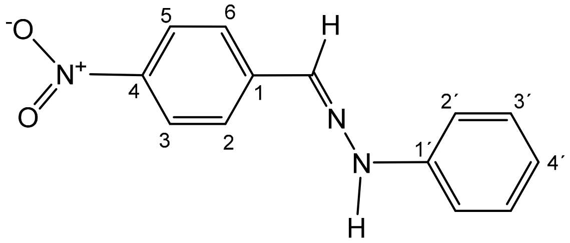


*(E)*-1-(4-nitrobenzylidene)-2-phenylhydrazine

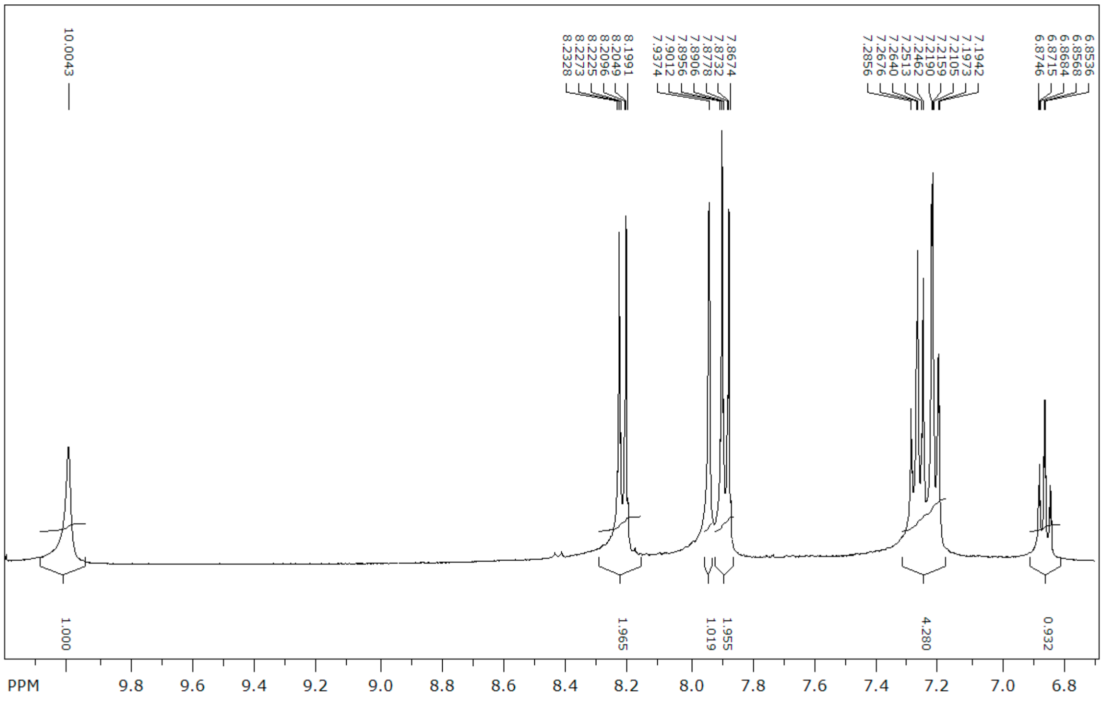


**Figure S9.** $^1$H-NMR for *(E)*-1-(4-nitrobenzylidene)-2-phenylhydrazine (compound **3**).

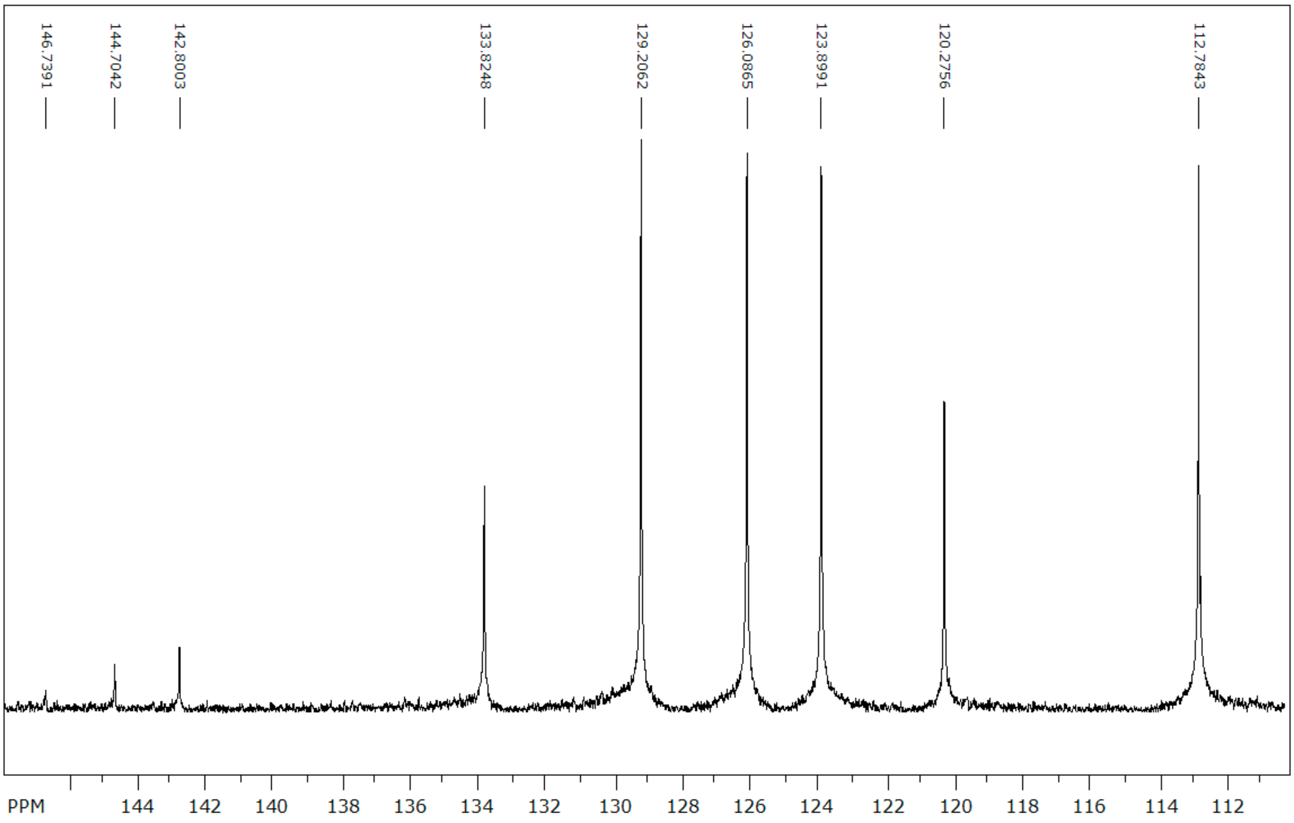


**Figure S10.** $^{13}$C-NMR for *(E)*-1-(4-nitrobenzylidene)-2-phenylhydrazine (compound **3**).

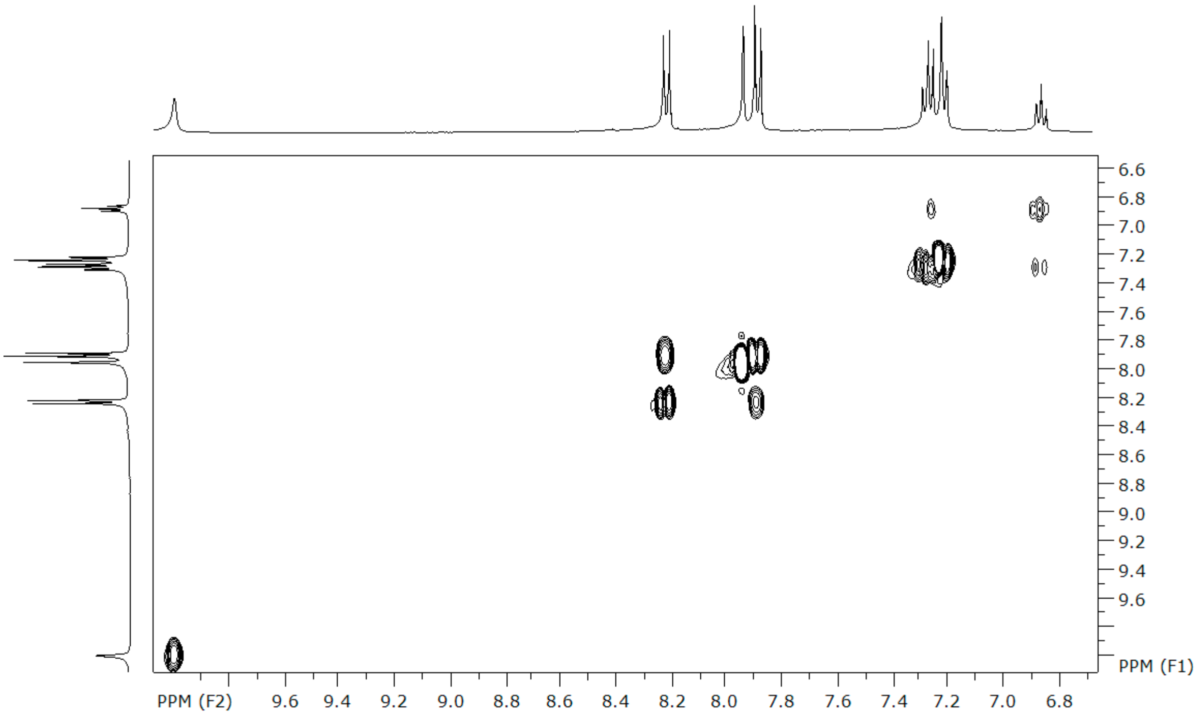


**Figure S11.** COSY for *(E)*-1-(4-nitrobenzylidene)-2-phenylhydrazine (compound **3**).

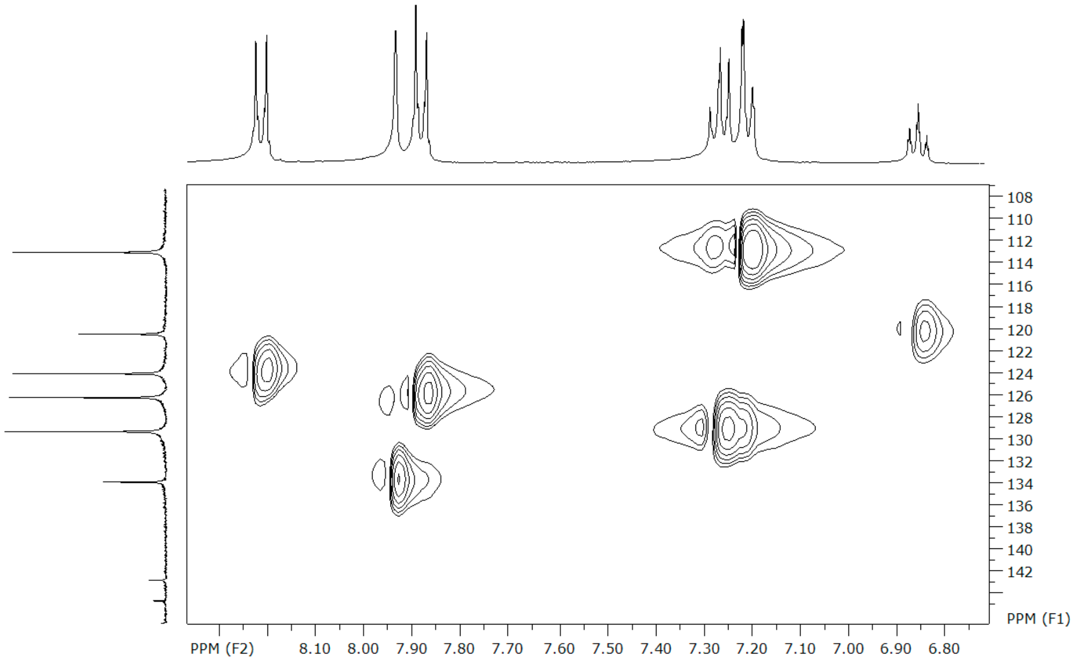


**Figure S12.** HSQC for *(E)*-1-(4-nitrobenzylidene)-2-phenylhydrazine (compound **3**).

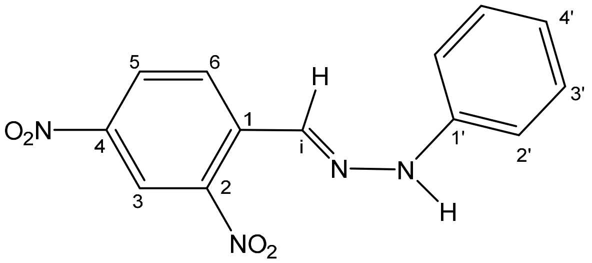


*(E)*-1-(2,4-dinitrobenzylidene)-2-phenylhydrazine

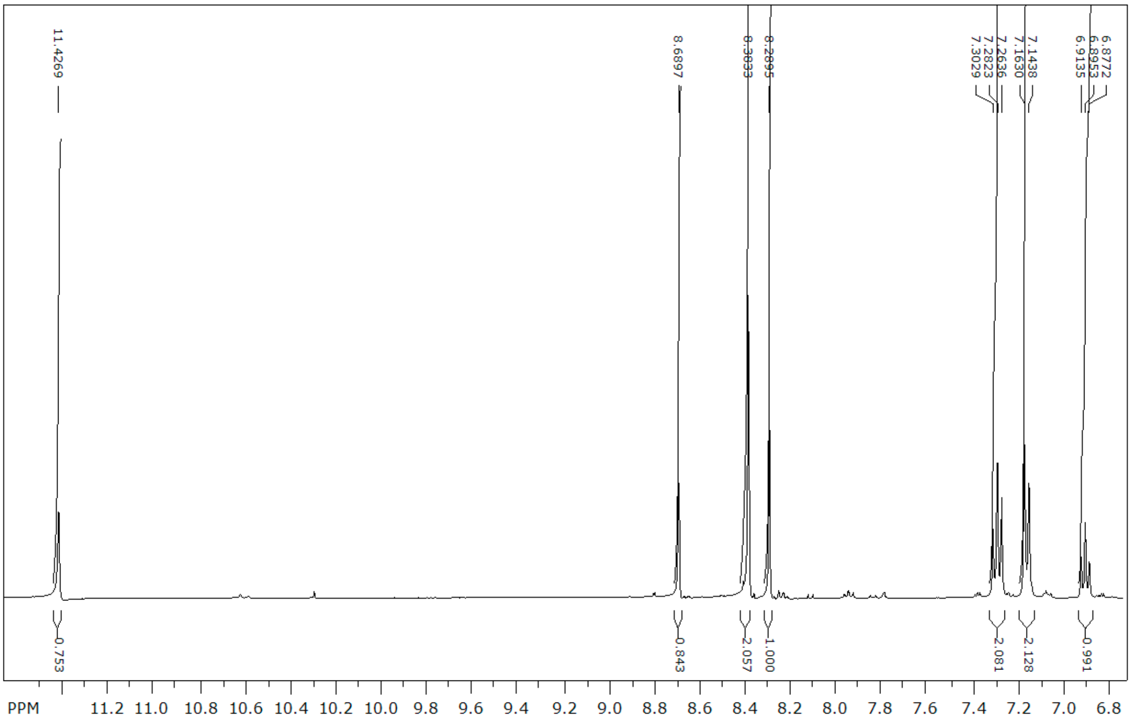


**Figure S13.** $^{1}$H-NMR for *(E)*-1-(2,4-dinitrobenzylidene)-2-phenylhydrazine (compound **4**).

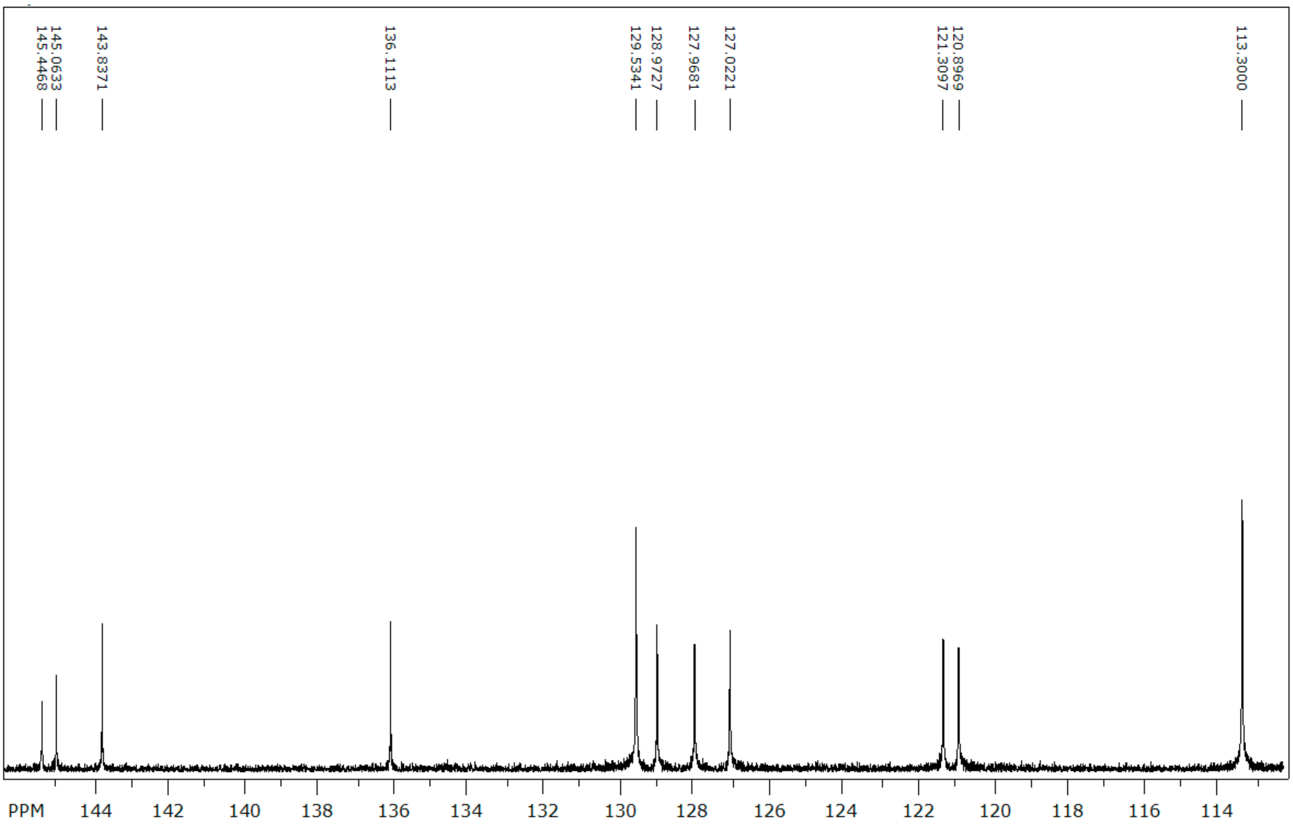


**Figure S14.** $^{13}$C-NMR for *(E)*-1-(2,4-dinitrobenzylidene)-2-phenylhydrazine (compound **4**).

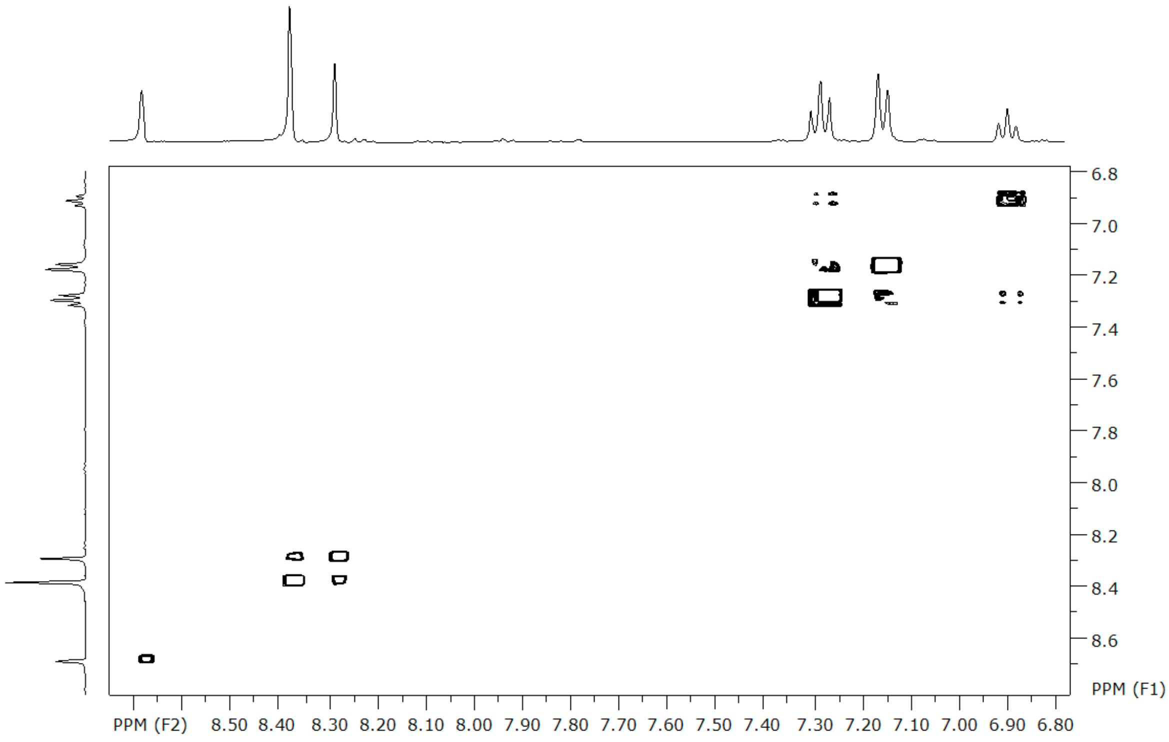


**Figure S15.** COSY for *(E)*-1-(2,4-dinitrobenzylidene)-2-phenylhydrazine (compound **4**).

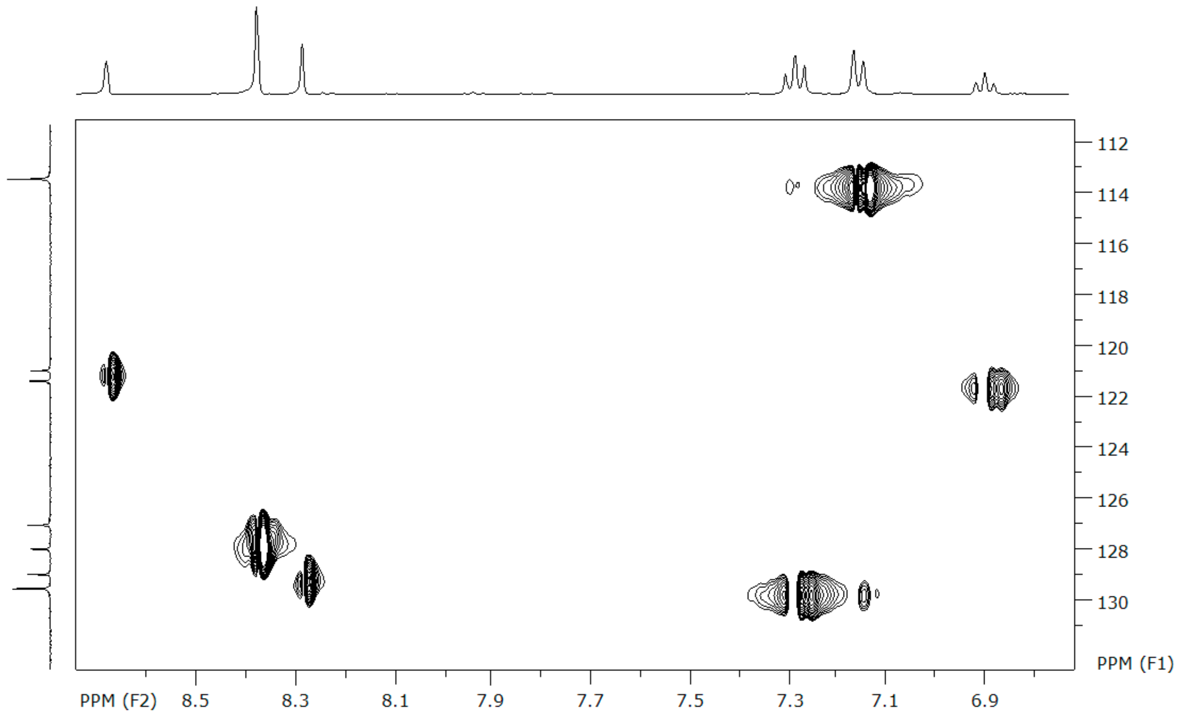


**Figure S16.** HSQC for *(E)*-1-(2,4-dinitrobenzylidene)-2-phenylhydrazine (compound **4**).
